# Optimizing the downstream MVA pathway using a combination optimization strategy to increase lycopene yield in *Escherichia coli*

**DOI:** 10.1186/s12934-022-01843-z

**Published:** 2022-06-20

**Authors:** Tao Cheng, Lili Wang, Chao Sun, Congxia Xie

**Affiliations:** 1 State Key Laboratory Base of Eco-Chemical Engineering, College of Chemistry and Molecular Engineering, Qingdao University of Science and Technology, No. 53 Zhengzhou Road, Qingdao, 266042 China; 2 Department of Pathology, the Affiliated Hospital of Qingdao University, Qingdao University, Qingdao, 266000 China; 3 CAS Key Laboratory of Bio-Based Materials, Qingdao Institute of Bioenergy and Bioprocess Technology, Chinese Academy of Sciences, No. 189 Songling Road, Laoshan District, Qingdao, 266101 China

**Keywords:** Lycopene, Mevalonate pathway, Ribosomal binding site library, *Escherichia coli*

## Abstract

**Background:**

Lycopene is increasing in demand due to its widespread use in the pharmaceutical and food industries. Metabolic engineering and synthetic biology technologies have been widely used to overexpress the heterologous mevalonate pathway and lycopene pathway in *Escherichia coli* to produce lycopene. However, due to the tedious metabolic pathways and complicated metabolic background, optimizing the lycopene synthetic pathway using reasonable design approaches becomes difficult.

**Results:**

In this study, the heterologous lycopene metabolic pathway was introduced into *E. coli* and divided into three modules, with mevalonate and DMAPP serving as connecting nodes. The module containing the genes (*MVK, PMK, MVD, IDI*) of downstream MVA pathway was adjusted by altering the expression strength of the four genes using the ribosome binding sites (RBSs) library with specified strength to improve the inter-module balance. Three RBS libraries containing variably regulated *MVK, PMK, MVD,* and *IDI* were constructed based on different plasmid backbones with the variable promoter and replication origin. The RBS library was then transformed into engineered *E. coli* BL21(DE3) containing pCLES and pTrc-lyc to obtain a lycopene producer library and employed high-throughput screening based on lycopene color to obtain the required metabolic pathway. The shake flask culture of the selected high-yield strain resulted in a lycopene yield of 219.7 mg/g DCW, which was 4.6 times that of the reference strain.

**Conclusion:**

A strain capable of producing 219.7 mg/g DCW with high lycopene metabolic flux was obtained by fine-tuning the expression of the four MVA pathway enzymes and visual selection. These results show that the strategy of optimizing the downstream MVA pathway through RBS library design can be effective, which can improve the metabolic flux and provide a reference for the synthesis of other terpenoids.

**Supplementary Information:**

The online version contains supplementary material available at 10.1186/s12934-022-01843-z.

## Introduction

Lycopene, a carotenoid with a red color and significant antioxidant effects due to its conjugated polyene structure [1], plays a vital role in human health. As an antioxidant, it exerts protection on the cardiovascular system by reducing the risk of myocardial infarction, lowering blood pressure, and preventing the oxidation of low-density lipoprotein cholesterol [2–4]. Additionally, research has shown that lycopene may be beneficial in preventing some types of malignant tumors, including prostate, lung, uterus and breast cancer [5, 6]. Furthermore, in synthetic biology, the lycopene enables high-throughput screening based on color variation and serves as an excellent model system for researching isoprenoids biosynthesis pathways [7]. Lycopene is currently directly extracted from natural resources [8]. Although the products extracted by this method are of high quality and have biological activity, the extraction method is relatively expensive due to the low content of lycopene in tomatoes and the complex multi-step process [9, 10]. Chemical synthesis has the advantage of low raw material cost, but it will bring health and food safety issues. Therefore, the microbial production of lycopene provides an attractive alternative because it can completely synthesize lycopene from cheap carbon sources, which has the potential to increase yield and sustainability and reduce production costs. Furthermore, as micro metabolic engineering and synthetic biology have progressed, it has become easier to modify, design, and optimize host microorganisms into advanced microbial cell factories [11–13]. *Escherichia coli*, as an excellent resident microorganism, is widely used for this purpose [14–17].

The mevalonate (MVA) pathway, which is native to eukaryotes and archaea, and the 2-C-methyl-D-erythritol 4-phosphate (MEP) pathway, which is native to most of the bacteria and plant plastids [18, 19], were employed to synthesize the precursors of lycopene isopentenyl diphosphate (IPP) and dimethylallyl diphosphate (DMAPP) during the biosynthesis process. In the MVA pathway, two molecules of acetyl-CoA are condensed to form 3-hydroxy-3-methylglutaryl-CoA (HMG-CoA), followed by a reduction reaction that generates mevalonate. After the production of mevalonate, two phosphates are added sequentially to the molecule by mevalonate kinase (MVK) and phosphomevalonate kinase (PMK). Finally, mevalonate-5-diphosphate is decarboxylated by mevalonate diphosphate decarboxylase (MVD) to form IPP. Once produced, IPP is converted reversibly to DMAPP by isopentenyl diphosphate isomerase (IDI). Subsequently, IPP and DMAPP were condensed to generate farnesyl diphosphate (FPP), which then was catalyzed by GGPP synthase, lycopene synthase, and lycopene desaturase, encoded by *Erg20*, *CrtE*, *CrtB,* and *CrtI,* respectively, to form geranylgeranyl diphosphate (GGPP, C20), colorless phytoene (C40), and red-colored lycopene.

With the advancement of synthetic biology technologies in recent years, the capacity to synthesis lycopene has been enhanced by introducing and optimizing heterologous metabolic pathways in *E. coli*. Due to the shortage of precursors in *E. coli* with native MEP pathway, the supply of IPP and DMAPP is usually enhanced by improving the MEP pathway or introducing a heterologous MVA pathway. Many studies have shown that introducing the exogenous MVA pathway into *E. coli* to improves IPP and DMAPP precursor supply. When the entire MVA pathway from *Streptomyces sp.* CL190 was introduced into *E. coli* with only a native MEP pathway, lycopene production increased by twofold [20]. Zhu et al. adopted a new target engineering strategy to optimize the MVA pathway in *E. coli* for improving the supply of precursor IPP and DMAPP, and the lycopene titer eventually reached 1.23 g/L in fed-batch fermentation [21]. In addition, Zhang et al. increased lycopene production 36-fold by altering the order of key enzymes crtE, crtB and crtI in the lycopene synthesis pathway [22], suggesting that a wrong gene arrangement can lead to a severe imbalance of enzymes in this pathway. Furthermore, ribosome-binding site libraries may be employed as a modifying approach to boost lycopene output, with the best strain producing 3.52 g/L in fed-batch fermentation [23].

In a previous study [24], we discovered that optimizing the ratios of the MVA pathway's five enzymes (*MVK\PMK\MVD\IDI\Isps4*) in vitro significantly improved the conversion efficiency of mevalonate to isoprene. However, due to the apparent intricacy of the cellular metabolic microenvironment, the results of in vitro are not appropriate to intracellular. Therefore, the development of synthetic biology methods can enable us to optimize the expression of the four genes of MVA in vivo to increase the metabolic flux of the MVA pathway. Manipulation of promoter, replication origin of plasmid, and RBS was thought to be the most straightforward approach for fine-tuning expression at transcriptional and translational levels [25]. Among them, RBS is essential for the translational control of enzyme activity [26]. It is preferable to fine-tune genes inside MPMI modules via RBS library engineering. RBS calculators have been designed to produce RBS sequences with precise intensities, which may then be used to build multi-enzyme pathways with great precision [27, 28]. As a result, further boosting lycopene synthesis necessitates fine-tuning of the complex metabolic networks that surround inter-and intra-modules and metabolic optimization at the transcriptional and translational levels.

**Fig. 1:**
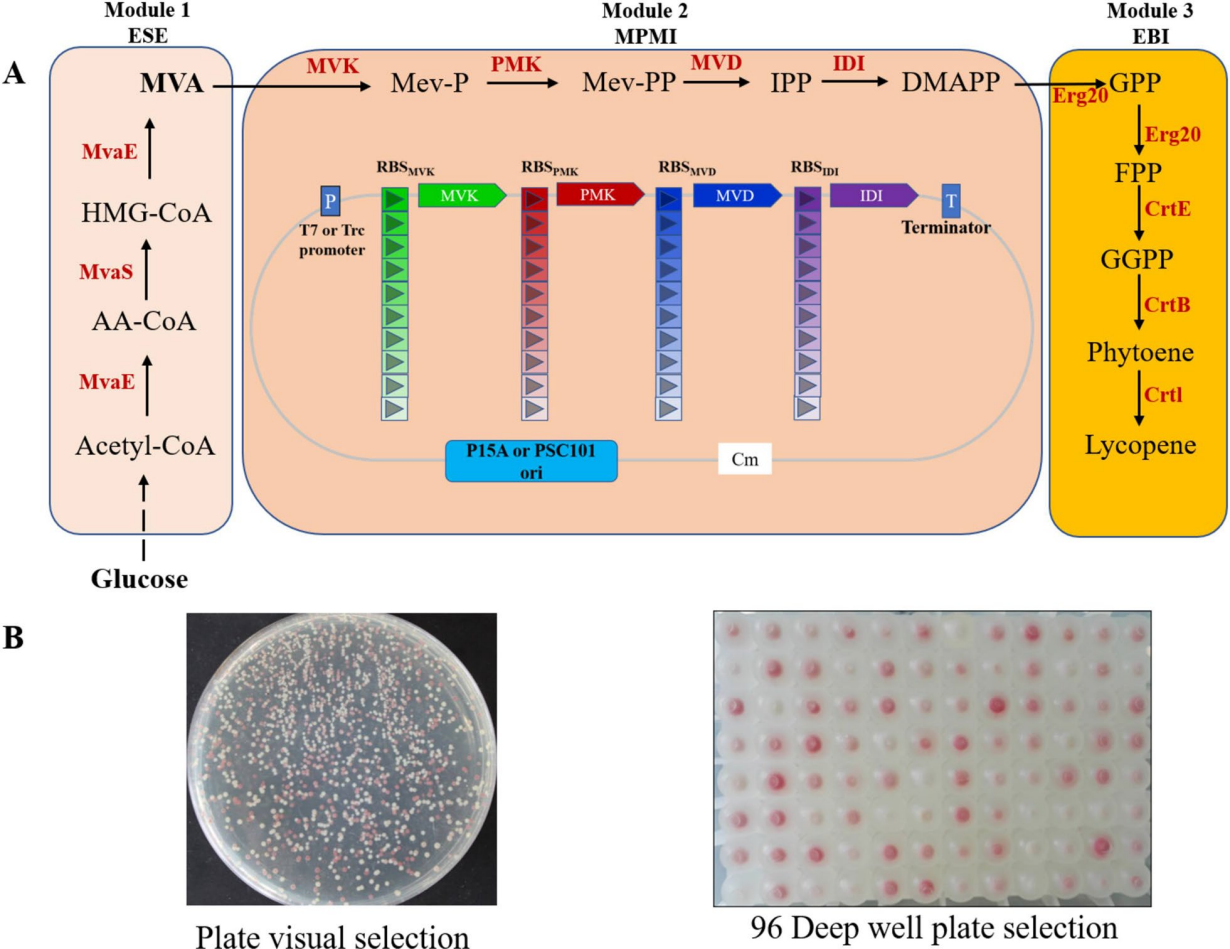
Library construction and visual selection based on-plate and 96 deep-well plate selections. **A** Lycopene accumulation pathway in *E. coli* by heterogenous MVA pathway. The whole pathway was divided to three modules: module ESE, the upstream MVA pathway; module MPMI, the downstream MVA pathway; module EBI, the lycopene synthesis pathway containing *Erg20*, *CrtE*, *CrtB,* and *CrtI*, In the module2, an RBS library and combinatorial constructs based on three plasmid backbones (with the pT7 promoter and the p15A origin, with pTrc promoter and p15A origin, or with pTrc promoter and pSC101) were employed to screen the optimal expression levels for the four genes MVK, PMK, MVD, and IDI to achieve the purpose of optimizing the lycopene metabolic flux. **B** On-plate and 96-deep-well plate visual selection of pHM-library. Each colony on the plate or 96-deep-well plate showed different colors indicating different lycopene accumulation

In this work, the capacity of microbial biosynthesis to be optimized by combining the coordinated expression of multiple genes in the module and the combinatorial approach between modules. To begin, the lycopene synthesis pathway was divided into three modules: module ESE and module MPMI and module EBI (Fig. 1A). Subsequently, the RBS library with defined strength for adjusting the expression levels of four genes in module MPMI was constructed based on different plasmid backbones with the variable promoter and replication origin using an oligo-linker mediated assembly (OLEM) method [26]. Finally, the RBS library was then transformed into engineered *E. coli* BL21 (DE3) /pCLES&pTrc-lyc to obtain a lycopene producer library and employed high-throughput screening based on lycopene color to obtain the required metabolic pathway. Finally, the MVA pathway obtained through screening increased lycopene yield, thus achieving the goal of "what you see is what you get."

## Results and discussion

### Mevalonate pathway optimization by RBS library construction

In the multienzyme pathway, the simple overexpression of one or more enzymes risks causing an imbalance between enzymes and upstream and downstream pathway modules, resulting in growth inhibition or poor yield owing to the cytotoxicity of accumulated intermediates, as well as the formation of new bottleneck nodes in the metabolic pathway [29, 30]. Liu et al. found that the upstream and downstream modules of the MVA pathway have severe metabolic imbalances, resulting in the output of mevalonate in the upstream module reaching about 84.0 g/L, while the production of isoprene, the product catalyzed by the downstream module, is only about 8.0 g/L [31]. The downstream module's four enzymes (MVK, PMK, MVD, IDI) and isoprene synthase were then expressed and purified in vitro using mevalonate as a substrate to catalyze isoprene synthesis. Adjusting the proportion of the five enzymes in vitro significantly improved the conversion efficiency of mevalonate to isoprene conversion [24]. The findings provided a novel ideal for optimizing the MVA pathway in *E. coli*: balancing the upstream and downstream modules by fine-tuning the expression levels of the four enzymes of the downstream module to enhance the metabolic flux. Lycopene is suitable for validating the high-throughput MVA metabolic pathway due to its vivid red hue.

The lycopene metabolic pathway was divided into three modules using mevalonate and DMAPP as connecting nodes. The module MPMI containing the genes (*MVK, PMK, MVD, IDI*) of downstream MVA pathway was adjusted by altering the expression strength of the four genes using the ribosome binding sites (RBSs) library with specified translation initiation rate (TIR), which was predicted by RBS calculator [27]. The semi-rational OLMA strategy was used to build an RBS library with defined strengths for each of the four genes *MVK, PMK, MVD,* and *IDI* [26]. This strategy can construct a library involving different variables such as promoters, RBSs, CDSs, and terminators in one step. The library is constructed based on three plasmid backbones: one medium copy (ori p15A) with strong promoter T7 (library HM, copy number 10–12, promoter T7), one medium copy (ori p15A) with medium strength promoter Trc (library MM, copy number 10–12, promoter Trc) and a low copy number plasmid (ori pSC101) with medium strength promoter Trc (library LM, copy number 1–2, promoter Trc). Each correctly assembled plasmid in the library contains four RBS randomly selected from four groups of RBS libraries. The library has a capacity of 10,000 combinations because each group has 10 different RBSs.

Three libraries of 10,000 combinations (HM, MM, and LM) were constructed using OLMA method (Fig. 1A). Then, the obtained library was co-expressed in *E. coli* with the upstream MVA pathway and the lycopene synthesis pathway and spread on the LB agar plate with inducers. After culturing for 72 h, red colonies with various intensities appeared on the plates (Fig. 1B), indicating their varying capacity for lycopene production. PCR analysis was performed on ten randomly selected red colonies of various intensities, and the gel electrophoresis revealed a 100% positive rate (Additional file 1: Fig S1). The results demonstrate that by assembling an RBS library and performing a simple color-based pre-screening, lycopene metabolic pathways with a diverse spectrum of metabolic fluxes can be obtained with minimal effort.

### High-throughput screening of recombinants with lycopene pathway

**Fig. 2:**
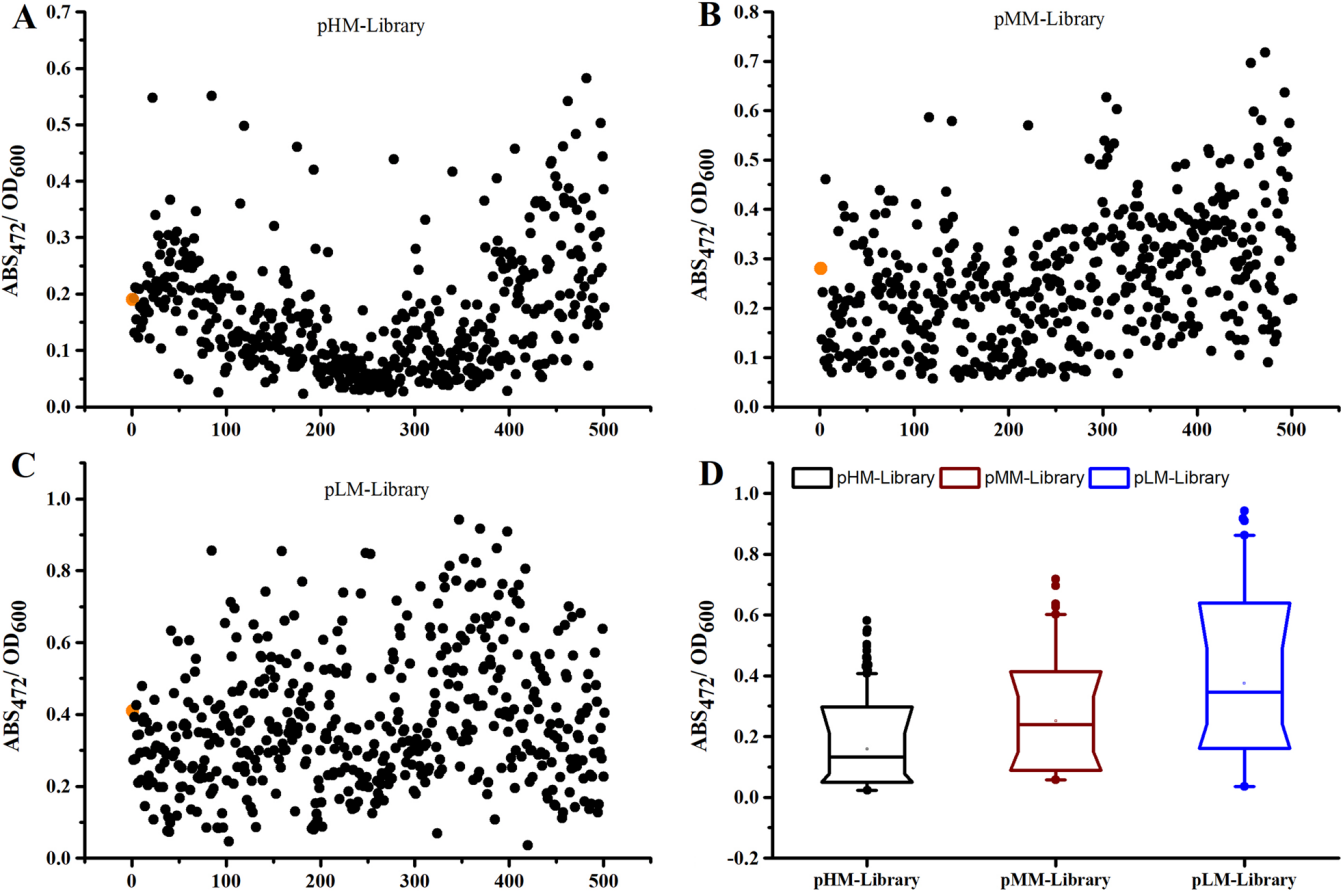
The results of 96-deep-well plate culture of 500 randomly picked strains in pHM-library, pMM-Library, and pLM-Library, respectively. The Y axis represents the absorbance of lycopene at 472 nm corrected using OD_600_. The orange dot represents reference strain. **A** The results of 96-deep-well plate culture of 500 strains in pHM-library. **B** The results of 96-deep-well plate culture of 500 strains in pMM-library. **C** The results of 96-deep-well plate culture of 500 strains in pLM-library. **D** Boxplots of the strains based on 500 randomly selected colonies per library

Colonies were chosen based on the depth of the colony color, which is a result of the color rendering of lycopene. In other words, the colonies grown on the LB-Glu solid medium exhibited a bright red hue compared to the colonies that accumulated little or no lycopene. 500 colonies from the pHM-library, 500 colonies from the pMM-library, and 500 colonies from the pLM-library, all of which were classified according to varying degrees of redness, were chosen using the visual selection method. All the selected colonies and three reference strains HM-CT, MM-CT, and LM-CT (positive control, which was a recombinant containing ESE, EBI, and MPMI modules with native RBSs under the three plasmid backbones) were grown in M9 medium on 96 deep-well plates. The culture medium showed various intensities of red after 96 h of cultivation (Fig. 1B), indicating the strains with different lycopene accumulation performance in the liquid medium. Lycopene was extracted from the samples with acetone and measured at 472 nm using absorbance and corrected with OD_600_. As shown in Fig. 2A–C, the relative lycopene production of the selected strains in 96-deep-well showed a 14 ~ 40-fold range (pHM-library 30-fold, pMM-library 14-fold, and pLM-library 40-fold), which indicated the combinatorial RBS library yield a large number of functional constructs with different lycopene production level.

**Fig. 3:**
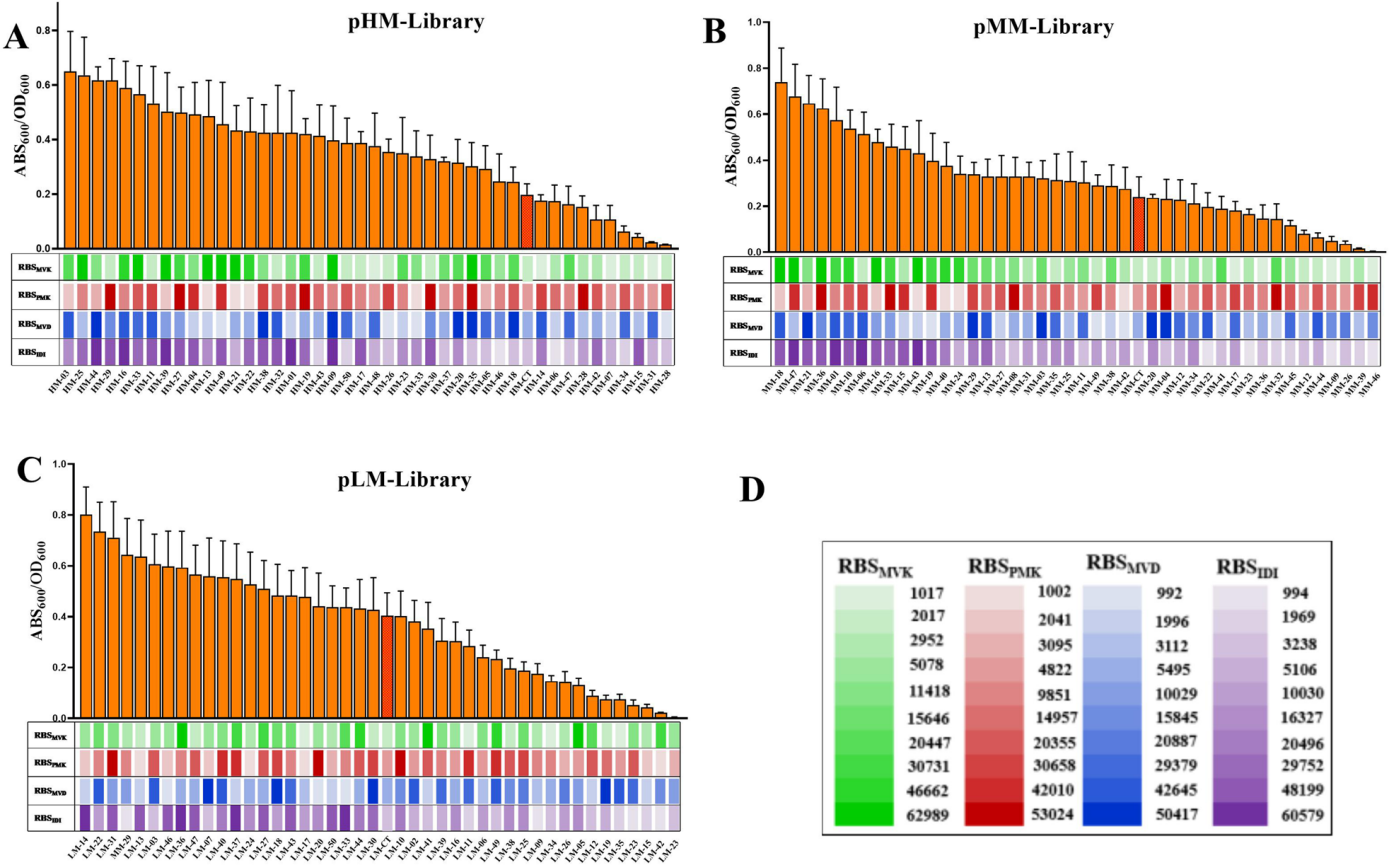
Characterization of the downstream MVA pathway (module2) activity and correlation between lycopene accumulation and RBS strengths. Lycopene accumulation was showed by orange columns. The reference strain with the native RBS was showed by red columns. RBS strengths were indicated by shades of color. **A** module MPMI activities and corresponding RBS strengths in pHM-library. **B** module MPMI activities and corresponding RBS strengths in pMM-library. **C** module MPMI activities and corresponding RBS strengths in pLM-library. **D** RBS strengths of RBS_mvk_ (green), RBS_pmk_ (red), RBS_mvd_ (blue), and RBS_idi_ (purple) indicated by shade of color. The results represent the means ± S.D. of three independent experiments

Furthermore, when the module ESE and module EBI were kept constant, the lycopene production was significantly increased by using the RBS library to fine-tune the four genes expression of the module MPMI (Fig. 3D). More than a quarter of the strains screened from the three libraries with various plasmid backbones were capable of producing more lycopene than the control strain. In the pHM-library, the best sample with ABS_472_/OD_600_ of 0.58 showed 3 times stronger than HM-CT and 30 times stronger than the lowest sample with ABS_472_/OD_600_ of 0.02. The highest one showed 2.5 times higher than MM-CT in the pMM-library and 14 times stronger than the lowest sample with ABS_472_/OD_600_ of `0.05. In the LM library, 50% of samples showed higher than LM-CT, the highest sample with AbS_472_/OD_600_ of 0.9, was higher 1.2-fold and 30 times than the LM-CT and the lowest sample, respectively. The LM library, which was under the plasmid backbone with the medium promoter and low copy, as shown in Fig. 3D, enables the screening of transformants with significant lycopene accumulation (ABS_472_/OD_600_ = 0.9). Low copy number and weaker promoters of the MVA downstream pathway operon were more favorable to lycopene accumulation, according to the findings (Fig. 2D). The explanation for this may be that decreasing the expression level of the midstream module can better match the upstream and the downstream module of lycopene synthesis, reducing the accumulation of intermediate and therefore increasing lycopene metabolic flux. Furthermore, this result revealed that altering enzyme activity generated by the strength of RBS has a substantial impact on the activity of the lycopene pathway. It was able to comprehensively investigate the activity of the MVA pathway since the RBS library constructed in this research caused a continuous shift in lycopene accumulation from zero to a high level (Fig. 2D). More significantly, this method can be used to synthesize additional terpenoids.

### Characterization of MVA pathway activities

To investigate the association between the RBS strengths and lycopene accumulation, 50 samples from each library were chosen and confirmed the RBS strengths. RBS_PMK_ and RBS_MVD_ have no apparent relationship with pathway activity, whereas RBS_MVK_ has a modest positive correlation with pathway activity, especially in the pHM-library and pMM-library. RBS_IDI_ had a significant positive correlation with pathway activity in the three libraries. As shown in Fig. 3A, B, the ten best-ranked strains of lycopene accumulation, eight strains in pHM-library and nine strains in pMM-library showed stronger TIR of RBS_MVK_, while all the strains showed stronger TIR of RBS_IDI_. Similarly, among the ten strains with the lowest lycopene production, seven strains in pHM-library and pMM-library showed weaker TIR of RBS_MVK_, eight strains showed weaker TIR of RBS_IDI_. IDI activity appeared to be critical for a high lycopene pathway. The findings were consistent with previous reports in which the MVK and IDI were identified as the crucial enzymes in the mevalonate pathway [32, 33]. Unfortunately, there was no clear proportion relationship involving these four genes, indicating that optimizing the MVA pathway by entirely rational design is challenging due to the sophisticated interactions among heterogeneous pathways and complex metabolic context. Given that there were 10,000 possible combinations, but only 500 colonies were selected for analysis, the small sample size resulted in the absence of a clear association between the RBS strengths and lycopene accumulation. Furthermore, the library developed in this study could be used to synthesize other products that could be selected via high-throughput visualization (colored products), such as carotene, astaxanthin, etc.

### Shake-flask fermentation of recombinants with selected pathways

**Fig. 4:**
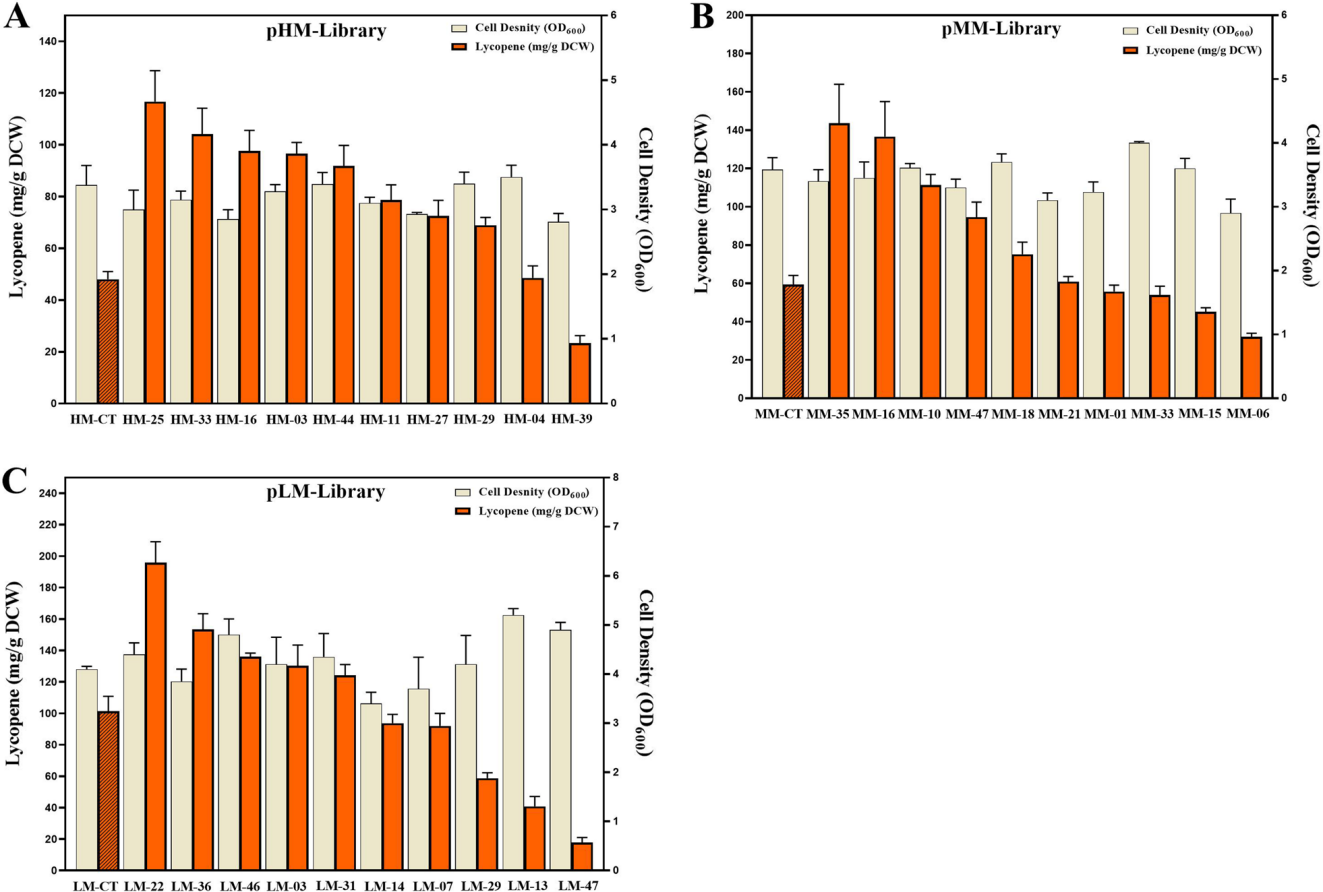
The lycopene production and biomass analysis. Ten best ranked samples selected from per library and their respective reference strains were further cultivated in shake flasks. Lycopene accumulation was showed by orange columns. The biomass was showed light yellow. **A** The lycopene accumulation and biomass of strains in pHM-library and of corresponding reference strain. **B** The lycopene accumulation and biomass of strains in pMM-library and of corresponding reference strain. **C** The lycopene accumulation and biomass of strains in pLM-library and of corresponding reference strain. The results represent the means ± S.D. of three independent experiments

For further validation in shake flask fermentation, the three reference strains and ten top-ranked samples from each of the three libraries were chosen. We begin by comparing the lycopene accumulate and cell density in the three reference strains grown in shake-flask. As shown in Fig. 4, the lycopene yield of the MM-CT strain, in which the operon of midstream module was initiated by medium strength promoter (Trc), was 60.4 mg/g DCW, which was barely 20% greater than that of HM-CT (48.3 mg/g DCW) with the stronger promoter (T7). However, as compared to MM-CT strain with the medium-copy plasmid (10 ~ 12), the lycopene production of the LM-CT strain harboring low-copy plasmid (1 ~ 2) increased the lycopene production by 100% to 90.2 mg/L DCW. The biomass of LM-CT is significantly higher than that of HM-CT and MM-CT. This result showed that at constant module ES and module EBI expression, lycopene production increased in lockstep with the decreasing strength of module MPMI. The trends of lycopene accumulation of reference strains in shake flasks were consistent with the results in 96-deep well.

Figure 4 shows that replacing RBSs with native ones increased lycopene yield by over 120 percent in all three libraries without changing any other sequence in the operon. Figure 4A shows that the sample pHM25 with the highest yield of 120 mg/g DCW in pHM-library was 250% stronger than the control strain HM-CT (48.3 mg/g DCW). In Fig. 4B, C, we found that the lycopene yield of sample MM35 (143.6 mg/g DCW) in MM-library and LM22 (219.7 mg/g DCW) in LM-Library increased by 140% and 120% compared to the reference strain MM-CT and LM-CT, respectively. The biomass of the LM-Library was significantly higher than that of the pHM-Library and pMM-library, which was consistent with the reference strain trend. While 70%-80% of the selected samples accumulated more lycopene than the control strain containing natural RBS in shake flask fermentation, 20% ~ 30% samples in each library perform worse than the control strains, which could be the inconsistency between high-throughput screening and shake-flask fermentation conditions. Over the course of many projects, strain productivity and growth rate may or may not improve when transferred from deep-well plates to shake flasks due to stochastic effects.

With constant upstream module and downstream module, as the promoter of the midstream module changed from pT7 to pTrc with decreasing strength, lycopene accumulation increased correspondingly. Furthermore, as the replication origin of the midstream module changed from p15A to pSC101 with reducing the copy number, lycopene production further improved likewise. The results demonstrate that the downregulation of the midstream module enhances the flux of the entire lycopene pathway, hence boosting product biosynthesis, which is consistent with previous research [34]. Excessive midstream module flux harmed lycopene accumulation and cell growth, which could be due to the downstream module's limited capacity or the potential cytotoxicity of accumulated intermediate metabolites like GPP and FPP [29, 35].

The four genes of the midstream module were fine-tuned based on these foundations by the construction of the RBS library, which further optimized the lycopene metabolic pathway and improved lycopene accumulation by 120% ~ 250% over the reference strains. In the case of limited samples (500 samples was selected in each library), the high-yielding lycopene (219.7 mg/g DCW) strain in shake flask fermentation in LM-library was screened. These results indicate that coordinating the expression of all the genes of the midstream module was an effective strategy for optimizing the lycopene synthesis pathway. Although the strain obtained through screening has a lower yield than the previously reported 448 mg/g DCW [36], a strain with increased lycopene accumulation can be created by increasing the screening quantity of the library. Supposing the screening of library samples is broadened, for example, by using the automated screening tool QPix 400 microbial colony pickers. In this case, it may be feasible to determine the relationship between the coordinate expression level of the four genes of the midstream module and the lycopene pathway flux. Simultaneously, we can screen for strains that produce color-producing terpenoid pigments like carotene, astaxanthin, and others by optimizing the RBS of the entire MVA pathway.

## Conclusion

The goal of this study was to use synthetic biology concepts to increase the efficiency of lycopene production. The one-step OLEM method, a semirational approach that can streamline the library creation procedure, was employed to construct three libraries containing RBS with varying intensities for each of four genes of the lycopene pathway based on various plasmid backbone. The visual selection of lycopene-accumulating colonies based on its vivid red hue simplified the subsequent screening steps even further in a glucose-rich medium plate. Following that, the association between RBS intensity and lycopene accumulation was evaluated in recombinant strains harboring various pathways. Although there was no clear proportion relationship between these four genes and lycopene metabolic flux, there was a difference in lycopene output between the ten best-ranked samples from each of the three libraries for shaker flask fermentation, ranging from 20.3 to 219.7 mg/g DCW. This finding suggests that it is feasible to balance the metabolic flux of the entire MVA pathway by leveraging a combination optimization strategy to optimize expression level of the downstream MVA pathway.

## Materials and methods

### Strains, media, and culture conditions

The *E. coli Trans* 5α chemically competent cells (Transgen Biotech, Beijing) were used for the plasmid and library construction. The *E. coli* BL21(DE3) chemically competent cells (Transgen Biotech, Beijing) were used as the host for lycopene production. *E. coli Trans* 5α and *E. coli* BL21(DE3) were routinely cultured in Liquid Luria–Bertani (LB) medium or LB agar plates with appropriate antibiotics at 37 °C. Strains and plasmids used in this study are listed in Table 1.

**Table 1:** Bacterial strains and plasmids used in this study

Strains / plasmid	Relevant genotype / property/sequence	Source / reference
Strains	Relevant genotype	
*E. coli* BL21(DE3)	F^−^ *ompT hsdS*_*B*_(*r*_*B*_^*−*^*m*_*B*_^*−*^) *gal dcm rne131* λ(DE3)	Transgen Biotec
*E. coli Trans* 5α	F-φ80 *lac* ZΔM15 Δ (lacZYA-*arg*F) U169 *end*A1 *rec*A1 *hsd*R17(rk^−^, mk^+^) *sup*E44λ-*thi*-1 *gyr*A96 *rel*A1 *pho*A	Transgen Biotec
HM-CT	*E. coli* BL21(DE3) containing pCLES, pHM-CT, and pTrc-lyc	This study
MM-CT	*E. coli* BL21(DE3) containing pCLES, pMM-CT, and pTrc-lyc	This study
LM-CT	*E. coli* BL21(DE3) containing pCLES, pLM-CT, and pTrc-lyc	This study

Plasmids	Property	
pAC-lyc	pACYDuet-1 derivative carrying genes *CrtE*, *CrtI*, *CrtB*	[34]
pCLES	pET28a ( +) derivative carrying genes *mvaE*, *mvaS,* T7 promoter, Kan^R^	[35]
pTrc-lyc	pTrchis2B derivative carrying genes *CrtE*, *CrtI*, *CrtB*	This study
pHM-CT	pACYDuet-1 derivative carrying genes *MVK*, *PMK*, *MVD*, *IDI*, T7 promoter, p15A ori, Cm^R^	This study
pMM-CT	Recipient plasmid with Trc promoter, derived from pACYDuet-1, carrying genes *MVK*, *PMK*, *MVD*, *IDI*, p15A ori, Cm^R^	This study
pLM-CT	Recipient plasmid with Trc promoter, derived from pACYDuet-1, carrying genes *MVK*, *PMK*, *MVD*, *IDI*, pSC101 ori, Cm^R^	This study
pHM-IDI	Recipient plasmid with T7 promoter, Golden gate ligation fragment for library construction and IDI ORF, p15A ori, CmR^R^	This study
pMM-IDI	Recipient plasmid with Trc promoter, derived from pACYDuet-1, Golden gate ligation fragment for library construction and IDI ORF, p15A ori, CmR^R^	This study
pLM-IDI	Recipient plasmid with Trc promoter, derived from pACYDuet-1, Golden gate ligation fragment for library construction and IDI ORF, pSC101 ori, CmR^R^	This study
pEA-MVK	Donor plasmid with MVK ORF and *Bsa* I sites, KanR^R^	This study
pEA-PMK	Donor plasmid with PMK ORF and *Bsa* I sites, KanR^R^	This study
pEA-MVD	Donor plasmid with MVD ORF and *Bsa* I sites, KanR^R^	This study

The single clone was picked and inoculated into a sterile 96-deep-plate containing 1 ML M9 minimal medium per well to visual-select the strain with different lycopene accumulation with appropriate antibiotic and 0.1 mmol IPTG. The microplate was grown at 37 °C for 24 h and then incubated at 30 °C for 48 h.

For the lycopene production in shake flasks, a single colony was inoculated into a 5 mL LB medium with appropriate antibiotics and incubated at 37 °C and 200 rpm overnight. Then, the 1 ml of seed culture was employed to inoculate into 250 mL shake-flasks containing 50 mL M9 minimal medium (15.3 g/L NaH_2_PO_4_·12H_2_O, 3 g/L KH_2_PO_4_, 1 g/L NH_4_Cl, 0.5 g/L NaCl) with 2.0% (w/v) glucose as the primary carbon source and cultivated at 37 °C and 200 rpm. When OD_600_ of culture reached about 0.8, 0.5 mM IPTG was added to induce protein expression. For lycopene production, the temperature was then lowered to 30 °C. After 48 h of culture, the cell mass and lycopene were measured.

## Plasmids and plasmid construction

All plasmids used in this study are described in Table 1. All primers used in this study are described in Additional file 1: Table S1. All PCRs were done using PrimerSTAR Max DNA polymerase (TAKARA, Dalian, China). Three donor plasmids (*pEA-MVK, pEA-PMK, and pEA-MVD*), a recipient plasmid *(pHM-IDI, pMM-IDI, or pLM-IDI*), and four groups of RBS with specific TIR were assembled in one reaction simultaneously for the construction of a combinatorial RBS library.

The Trc promoter operon and plasmid fragment were amplified by PCR from pTrcHis2B and pACYDuet-1, respectively. The two fragments were ligated using the p*EASY*-Blunt Zero Cloning Kit (Transgen Biotech, Beijing) to replace the T7 promoter, resulting in the recombinant plasmid pMM. A DNA fragment with replicon pSC101 and plasmid amplified from pCL1920 and pACYDuet-1 were ligated with the p*EASY*-Blunt Zero Cloning Kit to generate the recombinant plasmid pLM, in which the replicon p15A of pACYDuet-1 was replaced with pSC101. The MPMI operon containing the genes of *MVK*, *PMK*, *MVD*, and *IDI* was amplified from pYJM14 [37] by PCR with primers and cloned into the *Sal* I and *Sac* I sites of vector pACYDuet-1, pMM, and pLM to generate the recombinant plasmid pHM-CT, pMM-CT, and pLM-CT, respectively.

Three donor plasmids were constructed using the p*EASY*-Blunt Zero Cloning Kit. The gene of *MVK*, *PMK,* and *MVD* was amplified from pTrc-low by PCR and cloned into the plasmid p*EASY,* resulting in pEA-MVK, pEA-PMK and pEA-MVD, respectively. The restriction endonuclease *Bsa* I was introduced at both ends of the gene by primers.

The gene fragment of *IDI* containing the type IIS restriction endonuclease *Bsa* I in the 5’ was amplified from pYJM14 by primers to construct the recipient plasmid. Subsequently, the segment was ligated into an amplified pACYDuet-1 using the *pEASY*®-Uni Seamless Cloning and Assembly Kit (Transgen Biotech, Beijing), resulting in pHM-IDI. According to the same cloning method, a DNA fragment containing Trc promoter operon was amplified from pTrchis2B and cloned into an amplified pHM-IDI without promoter to generate pMM-IDI. Similarly, a DNA fragment with replicon pSC101 was ligated into an amplified pMM-IDI without replicon, yielding pLM-IDI.

### Construction of combinatorial RBS libraries

According to the requirements, the RBS sequence, which was evaluated and designed using RBS calculator [27, 28], with specific TIR ranging from 1000 to 60,000 for *MVK*, *PMK*, *MVD*, and *IDI* were synthesized in two reversed complemented single-strand oligos (Sangon Biotech, Shanghai), respectively. The detail of each RBSs oligos sequence with proper sticky-ends in the 5’ are described in Table 2. To generate double-strand oligos, the RBSs synthesized were dissolved in TE buffer (1 μM), and annealed at 95 °C for 5 min, then cooled to 4 °C at 0.1 °C/s. The double-stand RBS fragment was diluted 10 times and phosphorylated by T4 PNK (Thermo Fisher, USA). Finally, each group of phosphorylated RBS was mixed and employed to the further construction of the RBS library.

**Table 2:** RBS sequences for RBS libraries with defined TIR

RBS	ORF	T.I.R	Sequences (5’-3’)
MVK1	*MVK*	1017.7	TGCAAATTTATATCTACGTTAAAAATACTATCAAT
MVK2	*MVK*	2017	TGCAAATTTATATCTACGTTCGGAGCAACTTCAAT
MVK3	*MVK*	2952.5	TGCAAATTTATATCTACGTTCGTAGCGAGTTCAAT
MVK5	*MVK*	5078.99	TGCAAATTTATATCTACGTCAGAGTCGGGTTCAAT
MVK11	*MVK*	11418.02	TGCAAATTTATATCTACGTTATAAGGAAAGTCAAT
MVK15	*MVK*	15646.14	TGCAAATTTATATCTACGTTATAAGGAGCTTCAAT
MVK20	*MVK*	20447.37	TGCAAATTTATATCTACGTTATAAAGCAGGTCAAT
MVK30	*MVK*	30731.61	TGCAAATTTATATCTACGTTTAAAGGAACCTCAAT
MVK43	*MVK*	46662.15	TGCAAATTTATATCTACGTAAAGGAGCTTCTCAAT
MVK60	*MVK*	62989.43	TGCAAATTTATATCTACGTTTTAGGGAGAGTCAAT
PMK1	*PMK*	1002.56	AGGCCTCATAATAAATCAACATCACGGAGCGAGAA
PMK2	*PMK*	2041.67	AGGCCTCATAATAAATCAAGAAGGTCGGAGGAGAA
PMK3	*PMK*	3095.85	AGGCCTCATAATAAATCAATCAGGGGAGTAGAGAA
PMK5	*PMK*	4822.1	AGGCCTCATAATAAATCAAGTCCAGGGAGGGAGAA
PMK10	*PMK*	9851.05	AGGCCTCATAATAAATCAAGGGAGGTTACAGAGAA
PMK15	*PMK*	14957.6	AGGCCTCATAATAAATCAAGCTAAGGAGGAGAGAA
PMK20	*PMK*	20355.56	AGGCCTCATAATAAATCAAAGAACAGGAGGGAGAA
PMK30	*PMK*	30658.04	AGGCCTCATAATAAATCAAGTTAGAGGAGGGAGAA
PMK42	*PMK*	42010.79	AGGCCTCATAATAAATCAATAGTAAGGAGGGAGAA
PMK53	*PMK*	53024.08	AGGCCTCATAATAAATCAATAGAGGAGGTCGAGAA
MVD1	*MVD*	992.38	AGATTGAAAATTCCCAGACAGTCTCAGCCCGCACA
MVD2	*MVD*	1995.95	AGATTGAAAATTCCCAGACAAAATCGGATAGCACA
MVD3	*MVD*	3112.61	AGATTGAAAATTCCCAGACATAATTTCTAAGCACA
MVD5.5	*MVD*	5495.17	AGATTGAAAATTCCCAGACCCACTAAGCTCGCACA
MVD10	*MVD*	10029.99	AGATTGAAAATTCCCAGACCTAAGATTACAGCACA
MVD15	*MVD*	15844.55	AGATTGAAAATTCCCAGACTTAGGACCTTCGCACA
MVD20	*MVD*	20887.54	AGATTGAAAATTCCCAGACTCACAGAGGACGCACA
MVD30	*MVD*	29379.21	AGATTGAAAATTCCCAGACAGAAGGCAGCCGCACA
MVD43	*MVD*	42645.61	AGATTGAAAATTCCCAGACCGAGGATACCAGCACA
MVD50	*MVD*	50417.64	AGATTGAAAATTCCCAGACCCTAAGGCCCCGCACA
IDI1	*IDI*	993.72	AGAGTAAAAATAAGCCAAAACCCAAAGCAGCCTCA
IDI2	*IDI*	1969.19	AGAGTAAAAATAAGCCAAACGACGCTCAGACCTCA
IDI3	*IDI*	3238.36	AGAGTAAAAATAAGCCAAATCCTCACAGATCCTCA
IDI5	*IDI*	5106.49	AGAGTAAAAATAAGCCAAAGACACGAAGTTCCTCA
IDI10	*IDI*	10029.99	AGAGTAAAAATAAGCCAAACTAAGAAAACTCCTCA
IDI16	*IDI*	16327.19	AGAGTAAAAATAAGCCAAAACAGTAGGAACCCTC
IDI20	*IDI*	20496.44	AGAGTAAAAATAAGCCAAAATTAAAGAGATCCTCA
IDI30	*IDI*	29751.76	AGAGTAAAAATAAGCCAAATAAGGACTCTCCCTCA
IDI48	*IDI*	48198.92	AGAGTAAAAATAAGCCAAAGATAACGAGGTCCTCA
IDI60	*IDI*	60579.68	AGAGTAAAAATAAGCCAAAAAGGAGGAGAACCTCA

The Golden gate assembly method was employed to construct the RBS library [39]. 50 ng of recipient plasmids (pHM-IDI, pMM-IDI or pLM-IEI), 200 ng of donor plasmids (pEA-MVK, pEA-PMK, and pEA-MVE), 1.5 μl for each double-strand oligos (RBS_mvk_, RBS_pmk_, RBS_mvd_, RBS_idi_), *Bsa* I (0.5 1 μl, Thermo Fisher, USA), T4 DNA polymerase (2 μl, Thermo Fisher, USA), and 10 × T4 DNA polymerase buffer (2 μl) was mixed. Milli-Q water is added up to 20 μl. The hybrid solution was digested at 37 °C for 5 min, ligated for 10 min, cycled 15 times, then inactivated at 37 °C for 10 min, 50 °C for 5 min, and 80 °C for 5 min.

### Pathway assembly

Two microliter of the resultant reaction solution was transformed into the *E. coli* BL21(DE3) with the plasmid pCLES [38] of the upstream MVA pathway and the plasmid pTrc-lyc of the lycopene pathway to assemble the complete metabolic pathway of lycopene using glucose as a carbon source. Cells were spread on the LB plate with 2% glucose, appropriate antibiotics (34 μg/mL for chloramphenicol, 100 μg/mL for ampicillin and 100 μg/mL for kanamycin), and 0.1 mM IPTG. After incubation at 37 °C for 12 h, the plate was cultivated at room temperature for 48 h to accumulate lycopene for screening.

### Lycopene assay

#### Lycopene Assay for micro-plate cultivation

According to the color of colonies, 500 individual colonies were randomly selected from each library and inoculated into a sterile 96-deep-plate containing 1 ML M9 minimal medium per well with appropriate antibiotic and 0.1 mmol IPTG to induce the MVA pathway.

The microplate was grown at 37 °C for 24 h, and then incubated at 30 °C for 48 h. 200 μl of culture broth was transformed to another microplate for the biomass assay using Tecan Infinite M200 Pro at 600 nm. Dry cell weight (DCW) was calculated based on optical density at 600 nm (1 OD_600_ = 323 mg DCW/L). The plate was centrifuged using a centrifuge (Dynamic Scientific Ltd, Velocity 18R, England) at 4 °C and 4800 rpm (3118 rcf) for 10 min. The pellet was discarded and resuspended in 200 μl of pure water, and added 600 μl acetone, which acted as an extraction solution. Subsequently, lycopene accumulated in the cell was extracted by incubation at 50 °C for 30 min. A glass microplate with 200 μl of the red-colored supernatant was used to measure absorbance at 472 nm. The Tecan Infinite M200 Pro was used to create a standard curve by measuring the OD_472_ of commercial lycopene at various concentrations.

#### Lycopene Assay for Shake flask cultivation

The cells from 1 ml of culture were collected by centrifuge at 13,000 g and 4 °C for 2 min and resuspended with 200 μl of sterile water, then added 800 μl of acetone and vortex shock for 2 min. The extraction solution was centrifuged as described above and transformed to a glass micro-plate for absorbance measurement at 472 nm.

The biomass of the cultures was determined by measuring optical density at 600 nm using a spectrophotometer (Cary 50 UV–vis, Varian). Cell density samples were diluted as necessary to fall within the linear range.

## Supplementary Information

**Additional file 1: Table S1**. Primers used in this study. **Figure S1**. Ten colonies were randomly picked and analyzed by colony PCR to determine ratio of successfully assembled pHM-library, pMM-library, and pLM-library. M marker.

## Data Availability

The datasets supporting the conclusions of this article are included within the article.

## Abbreviations

*MvaE*: Acetyl-CoA acetyltransferase/HMG-CoA reductase
*MvaS*: HMG-CoA synthase
*MVK*: Mevalonate kinase
PMK: Phosphomevalonate kinase
*MVD*: Mevalonate pyrophosphate decarboxylase
*IDI*: IPP isomerase
*Erg20*: Farnesyl/geranyl pyrophosphate synthase
*CrtE*: Geranylgeranyl diphosphate synthase
*CrtB*: Phytoene synthase
*CrtI*: Phytoene desaturase
AA-CoA: Acetoacetyl-CoA
HMG-CoA: 3-Hydroxy-3-methylglutaryl-CoA
Mev-P: Mevalonate 5-phosphate
Mev-PP: Mevalonate 5-diphosphate
IPP: Isopentenyl pyrophosphate
DMAPP: Dimethylallyl pyrophosphate
FPP: Farnesyl pyrophosphate;
GGPP: Geranylgeranyl pyrophosphate
