# Correction to: Optimizing the downstream MVA pathway using a combination optimization strategy to increase lycopene yield in *Escherichia coli*

**DOI:** 10.1186/s12934-022-01927-w

**Published:** 2022-10-08

**Authors:** Tao Cheng, Lili Wang, Chao Sun, Congxia Xie

**Affiliations:** 1 State Key Laboratory Base of Eco-Chemical Engineering, College of Chemistry and Molecular Engineering, Qingdao University of Science and Technology, No. 53 Zhengzhou Road, Qingdao, 266042 China; 2 Department of Pathology, the Affiliated Hospital of Qingdao University, Qingdao University, Qingdao, 266000 China; 3 CAS Key Laboratory of Bio-Based Materials, Qingdao Institute of Bioenergy and Bioprocess Technology, Chinese Academy of Sciences, No. 189 Songling Road, Laoshan District, Qingdao, 266101 China

## Correction to: Microbial Cell Factories (2022) 21: 121 https://doi.org/10.1186/s12934-022-01843-z

Unfortunately, the Supplementary file was originally published with incorrect equal contribution statement which should be removed. The corrected Supplementary file (Additional file 1) is available in the below link. The original article [1] has been corrected.

## Supplementary Information

**Additional file 1: Table S1.** Primers used in this study. **Figure S1**. Ten colonies were randomly picked and analyzed by colony PCR to determine ratio of successfully assembled pHM-library, pMM-library, and pLM-library. M marker.

## References

[1] Cheng T, Wang L, Sun C, Xie C (2022). Optimizing the downstream MVA pathway using a combination optimization strategy to increase lycopene yield in *Escherichia coli*. Microb Cell Factories.

