# Supplementary material for: Optimizing the downstream MVA pathway using a combination optimization strategy to increase lycopene yield in Escherichia coli
Source: Microb Cell Fact. 2022 Jun 20;21:121. doi: 10.1186/s12934-022-01843-z (PMC9208136; doi:10.1186/s12934-022-01843-z)
Supplement: Supplementary file 1 — Additional file 1: Table S1. Primers used in this study. Figure S1. Ten colonies were randomly picked and analyzed by colony PCR to determine ratio of successfully assembled pHM-library, pMM-library, and pLM-library. M marker. [file 12934_2022_1843_MOESM1_ESM.docx]

Additional file 1

Optimizing the downstream of MVA pathway using a combination optimization strategy to increase lycopene yield in *Escherichia coli*

Tao Cheng ^1, 3, #, *^, Lili Wang^2, #^, Chao Sun ^1, 3^, Congxia Xie ^1, *^

^1^State Key Laboratory Base of Eco-Chemical Engineering, College of Chemistry and Molecular Engineering, Qingdao University of Science and Technology, Qingdao 266042, China

^2^Department of Pathology, the Affiliated Hospital of Qingdao University, Qingdao University, Qingdao 266000, China

^3^CAS Key Laboratory of Bio-based Materials, Qingdao Institute of Bioenergy and Bioprocess Technology, Chinese Academy of Sciences, Qingdao 266101, China

# The first two authors contributed equally to the work

*Corresponding author.

Tao Cheng, No. 189 Songling Road, Laoshan District, Qingdao Institute of Bioenergy and Bioprocess Technology, Qingdao, China 266101. Tel: +86 532 80662681. mail address: chengtao@qibebt.ac.cn

Congxia Xie, No. 53 Zhengzhou Road, College of Chemistry and Molecular Engineering, Qingdao University of Science and Technology, Qingdao, China 266042. Tel: +86 532 84023927. Email address: xiecongxia@126.com

Table S1: Primers used in this study

| Primers | Sequence (5’ to 3’) |  |
| --- | --- | --- |
| BsaI_IDI_F | CCGCCAGTAGAGGTCTCG CTGCCGACAACAATAGTATG |  |
| IDI_R | CTGTTCGACTTAAGCATTAT TTATAGCATTCTATGAATTT |  |
| pACY_F | ATAATGCTTAAGTCGAACAG |  |
| pACY_R | CGAGACCTCTACTGGC GGGGTCTCAAAGTTAAACAAAATTATTT |  |
| MVK_F | ATGTCATTACCGTTCTTAAC |  |
| MVK_R | TTATGAAGTCCATGGTAAATTCG |  |
| PMK_F | ATGTCAGAGTTGAGAGCCTT |  |
| PMK_R | TTATTTATCAAGATAAGTTTCCGG |  |
| MVD_F | ATGACCGTTTACACAGCATC |  |
| MVD_R | TTATTCCTTTGGTAGACCAG |  |
| CrtE_F | AAAGGCACAGCGTCTCATGCTT |  |
| CrtI_R | ATCTACCACCTGCGTGCGCT |  |
| pTrc_F | AGCGCACGCAGGTGGTAGAT TAAACGGTCTCCAGCTTGGC |  |
| pTrc_R | AAGCATGAGACGCTGTGCCTTT CTCATTTCAGAATATTTGCC |  |


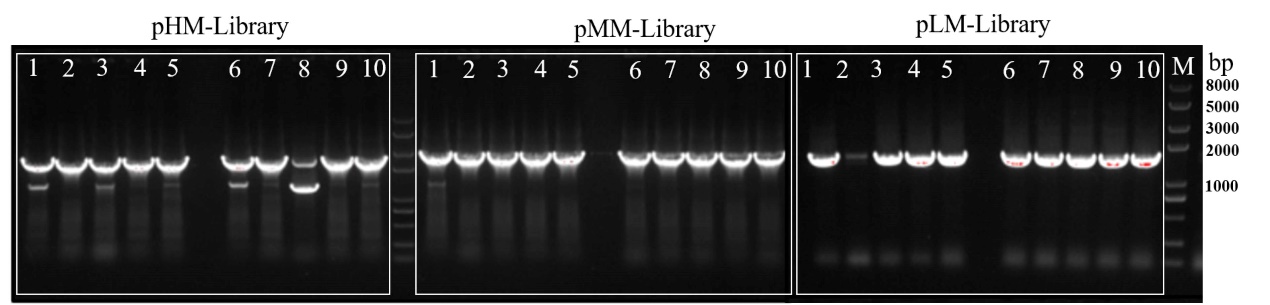


Figure S1 Ten colonies were randomly picked and analyzed by colony PCR to determine ratio of successfully assembled pHM-library, pMM-library, and pLM-library. *M* marker.
